# Application of magnetically actuated self-clearing catheter for rapid in situ blood clot clearance in hemorrhagic stroke treatment

**DOI:** 10.1038/s41467-022-28101-5

**Published:** 2022-01-26

**Authors:** Qi Yang, Ángel Enríquez, Dillon Devathasan, Craig A. Thompson, Dillan Nayee, Ryan Harris, Douglas Satoski, Barnabas Obeng-Gyasi, Albert Lee, R. Timothy Bentley, Hyowon Lee

**Affiliations:** 1 Weldon School of Biomedical Engineering, Purdue University, West Lafayette, IN 47907 USA; 2 Center for Implantable Devices, Purdue University, West Lafayette, IN 47907 USA; 3 Birck Nanotechnology Center, Purdue University, West Lafayette, IN 47907 USA; 4 School of Electrical and Computer Engineering, Purdue University, West Lafayette, IN 47907 USA; 5 College of Veterinary Medicine, Purdue University, West Lafayette, IN 47907 USA; 6 Goodman Campbell Brain and Spine, Indianapolis, IN 46202 USA

**Keywords:** Translational research, Biomedical engineering, Cardiovascular diseases

## Abstract

Maintaining the patency of indwelling drainage devices is critical in preventing further complications following an intraventricular hemorrhage (IVH) and other chronic disease management. Surgeons often use drainage devices to remove blood and cerebrospinal fluid but these catheters frequently become occluded with hematoma. Using an implantable magnetic microactuator, we created a self-clearing catheter that can generate large enough forces to break down obstructive blood clots by applying time-varying magnetic fields. In a blood-circulating model, our self-clearing catheters demonstrated a > 7x longer functionality than traditional catheters (211 vs. 27 min) and maintained a low pressure for longer periods (239 vs. 79 min). Using a porcine IVH model, the self-clearing catheters showed a greater survival rate than control catheters (86% vs. 0%) over the course of 6 weeks. The treated animals also had significantly smaller ventricle sizes 1 week after implantation compared to the control animals with traditional catheters. Our results suggest that these magnetic microactuator-embedded smart catheters can expedite the removal of blood from the ventricles and potentially improve the outcomes of critical patients suffering from often deadly IVH.

## Abstract (web summary)

The authors present a magnetically powered, self-clearing implantable catheter that can rapidly remove hematoma from the brain without using drugs. Animals treated with this device had significantly better survival following a hemorrhagic stroke compared with regular catheters.

## Introduction

Brain hemorrhage is one of the most common and lethal forms of stroke, affecting more than 2 million patients annually worldwide. In 45% of these cases, bleeding occurs inside the ventricles of the brain and leads to intraventricular hemorrhage (IVH). When the blood clot (hematoma) forms and obstructs the circulation of cerebrospinal fluid (CSF), IVH can lead to an even deadlier condition (50–80% mortality) known as post-hemorrhagic hydrocephalus (PHH, 40% of IVH)^1,2^. IVH is especially common in preterm pediatric patients with low birth weight, and subsequent PHH can have devastating neurodevelopmental consequences^3^.

A gold standard in the treatment of IVH is the rapid removal of hematoma to prevent further deterioration using various interventional methods including open surgery, catheter-based drainage, and thrombolytic agents. These are used to prevent the mass effect (i.e., displacement of neural tissue), relieve the elevated intracranial pressure (ICP), restore CSF flow, and minimize blood exposure to the ventricles^4–6^. However, using thrombolytic agents on hemorrhagic patients is controversial due to the elevated risk of additional bleeding^7^. A recently completed large-scale clinical trial for small molecule interventions (e.g., tissue plasminogen activator) has not demonstrated adequate benefit for IVH patients^8^.

For some IVH patients, external ventricular drainage (EVD), ventricular reservoir devices, or neuroendoscopy are used to remove the blood-filled CSF and stabilize increasing ICP^4,9^. Much like the use of thrombolytic agents, the clinical efficacy of these surgical interventions are not very clear and there is no established guideline to help determine the proper condition for EVD implantation^6^. The thrombolytic agents and the EVDs are typically used only after the bleeding has stopped and the size of the hematoma has decreased below a threshold, which can prolong the blood exposure and the mass effect.

This may partly be due to the difficulty in maintaining the patency of the drainage device in the blood-filled ventricle^10,11^. To facilitate blood clot removal, thrombolytic agents are used in conjunction with a drainage device for increased clot resolution^12^. However, using fibrinolytic agents on hemorrhagic patients is a controversial topic due to the elevated risk of additional bleeding and their unknown long-term effects^7,13^.

The obstruction of drainage devices is a notorious issue recognized for more than half a century^14^. To restore patency in obstructed drainage systems, catheter flushing often takes place in intensive care units^15^. However, flushing alone is often ineffective in clearing the hematoma, which leads to several replacements of failed drainage devices that also increase the risk of infection and other complications including ventriculitis^16–18^. The high obstruction rate of these critical medical devices results in significant complications when treating IVH, PHH, and other applications where indwelling catheters are needed^13^.

There are ongoing efforts to combat the occlusion issues in EVD and other drainage devices. For example, researchers have investigated the use of dual catheters in high volume IVH^19,20^. There are also reports of using high-intensity focused ultrasound as a way to dissolve clots in situ^21–24^. However, increasing the number of catheters also increases the risk of infection and the potential concerns of off-target tissue damage remain for the ultrasound-induced clot reduction.

Magnetic microactuators have previously been shown to be effective in vitro in removing molecular and cellular scale biofouling materials on-demand using a remote application of time-varying magnetic fields^25–28^. These devices have been shown to have good mechanical robustness against static and dynamic stresses due to magnetic resonance (MR) imaging or fatigue-induced failures^27^. Moreover, there are now examples of magnetically actuated microrobots for various in vivo clinical applications including drug delivery^29–32^, stem cell transplantation^33^, and minimally invasive surgeries^34–36^. These magnetically powered micro- and nano-scale transducers provide a significant advantage over conventional medical devices because they allow implanted devices to be manipulated in situ without the need for additional surgical intervention using externally applied magnetic fields^37^.

In this work, we present a novel self-clearing implantable catheter that is enabled by microscale magnetic actuators. These magnetic microactuators are externally controlled via time-varying magnetic fields from a rotating permanent magnet or a bespoke electromagnet and can rapidly break down intraventricular thrombosis with its large-deflection actuation to maintain patency in implantable catheters. Using a series of in vitro and in vivo experiments, here we show that these self-clearing smart catheters with magnetic microactuators can help reduce the size of the obstructive hematoma, improve drainage device reliability, and increase the survival of IVH-induced animals.

## Results

### Device design and characterization

**Fig. 1:**
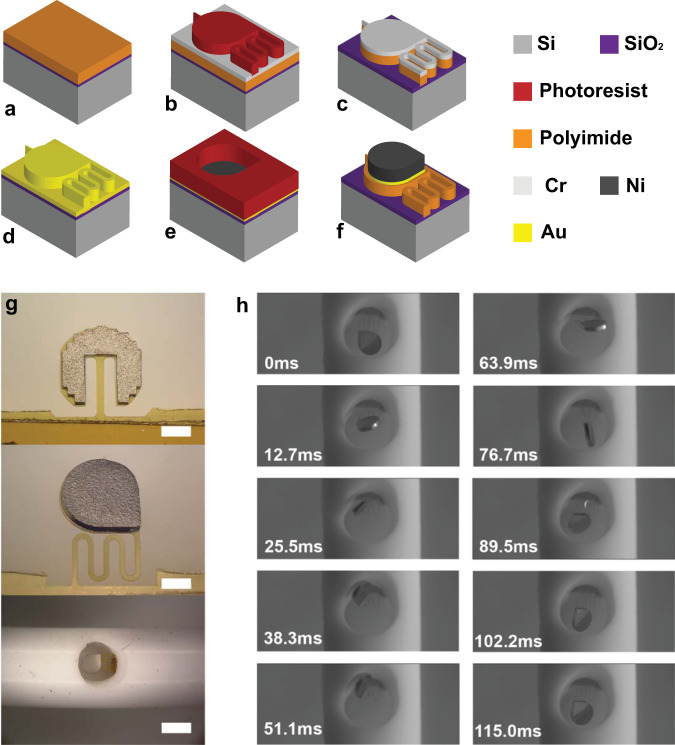
Design and fabrication of a self-clearing ventricular catheter. **a** Polyimide spin coat and curing on single-crystal silicon (Si) wafer with silicon dioxide (SiO_2_) release layer. **b** Evaporation of Cr etching mask and define photoresist for actuator outline. **c** Wet etch of chromium (Cr) and dry etch of polyimide. **d** Removal of Cr and evaporation of gold (Au) as a conduction layer. **e** Application of photoresist and nickel (Ni) electroplating. **f** Removal of photoresist and remaining Au layer. **g** Optical images of fabricated magnetic actuators of straight type (top) and serpentine type (middle). Scale bars = 325 μm. A catheter with an integrated actuator (bottom). Scale bar = 1 mm. **h** A typical motion of actuation in deionized (DI) water for one actuation period.

To fabricate the implantable magnetic microactuator for the prevention of hematoma accumulation, we applied conventional surface micromachining techniques as described in the Methods section. Figure 1a–f briefly shows the fabrication process. Figure 1g highlights the two microactuator designs. The dimensions of each design are listed in Supplementary Tables 1 and 2. The first design is a simple rectangular cantilever, which has been demonstrated to have a good mechanical robustness^27,38^.

The new design features a serpentined flexure with tear-drop-shaped ferromagnetic elements (Fig. 1g). The serpentine flexure consists of 4 windings with 5, 400 μm-long straight segments connected by 4 arcs providing 100 μm gaps in between them. Serpentine flexures have had pervasive use in the MEMS field and have been reported for making a number of ultra low-frequency resonators and piezoelectric energy harvest devices^39–43^. In our case, the serpentine flexure provides a 6-times smaller bending stiffness and enhanced deflection within a limited footprint. In addition to improved beam design, the large aspect ratio magnetic elements provide additional magnetic torque compared to the previous versions despite having nearly identical volume because of magnetic geometry anisotropy. A detailed description of the magnetic element dimensions and the analytical evaluation of magnetic torque is in the Supplementary Discussion. The microfabricated thin-film microactuators were then integrated into a custom catheter to create a self-clearing ventricular catheter (Fig. 1g). Figure 1h illustrates a typical actuation motion of a microactuator with serpentine flexure (Supplementary Movie 1). In the presence of time and spatially varying magnetic fields, the actuator deflects in and out of the plane for a more dynamic hematoma removal (Supplementary Movie 2). Dynamically, the actuator featured the largest actuation amplitude near 10–20 Hz in water (Supplementary Fig. 3, Supplementary Movie 3).

**Fig. 2:**
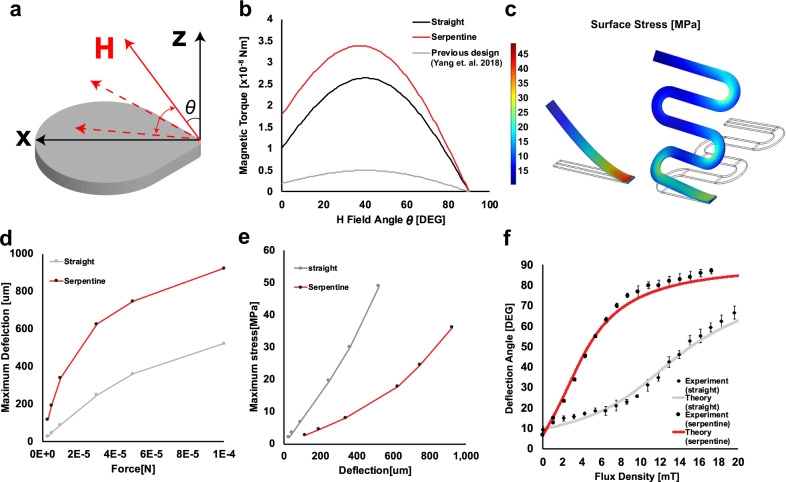
Mechanical characterization. **a** Definition of the coordinate system (*x*–*z*) and magnetic field (*H*) angle *θ*. **b** Calculated magnetic torque produced on the ferromagnetic element of serpentine (red line) and straight actuator (black line) under 15 mT at different field angles. **c** Finite element analysis of stress distribution on cantilevers’ surfaces under 0.1 mN load at the tip. **d** Maximum calculated deflection under loading condition (0.01–0.1 mN) for both cantilever types from finite element simulation. **e** Maximum calculated stress under various deflection conditions for both cantilever types from finite element simulation. **f** Static deflection angle prediction and measurements for the serpentine and the straight flexures. Each data point represents a different experiment using an independent sample. Data are expressed as mean ± standard deviation (*n* = 3).

Figure 2 demonstrates the improvements of the new magnetic microactuator over the straight beam design. We characterized the magnetic properties of the electroplated ferromagnetic elements using a magnetometer to calculate the magnetic torque assuming a field-dependent, non-saturated magnetization Supplementary Figs. 1–3). Figure 2a, b shows that the magnetic torque varies as the function of applied magnetic field angle *θ* and that maximum torque can be achieved at 40°. We saw as much as 75% improvement when *θ* = 40°.

We found that the new flexure design improved actuator displacement per applied magnetic field strength. The numerical analysis showed a larger deflection and smaller stress on the serpentine flexure than the straight beam when a vertical point load was applied to its tip (Fig. 2c). Figure 2d, e shows varying degrees of deflection and corresponding maximum stress on the beam at various load ranges. We saw that the serpentine flexure provides twice as much displacement over the straight beam over the same loading condition. The maximum static stress calculated for serpentine flexure was 36 MPa at 0.1 mN, which is significantly lower than the tensile stress of this polyimide (131 MPa).

The 0.1 mN loading was chosen from the MR safety perspective^26^. A typical human whole-body MR system produces a maximum spatial gradient between 10 and 50 mT/m^44–46^. Assuming the ferromagnetic element was fully saturated, the 0.1 mN load corresponds to the magnetic force produced from a gradient of 4 T/m, an order of magnitude greater than the physical constraint. Therefore, it is unlikely that our device will be damaged due to mechanical deformation in an MR system.

The improvements in magnetic torque and mechanical compliance of the flexure allow the new microactuator to achieve a greater deflection per given magnetic flux density (Fig. 2f). Although the previous microactuators were effective against protein and cellular biofouling^27,47^, those were not effective against macroscopic hematoma. Therefore, our primary objective was to increase the magnetic torque and rapid displacement to better remove a more robust thrombotic mass that plagues drainage devices in IVH patients. Our results show that we were able to achieve this goal. The new microactuator achieved >80° deflection with 15 mT compared to only 30° for the previous design.

### In vitro evaluation

**Fig. 3:**
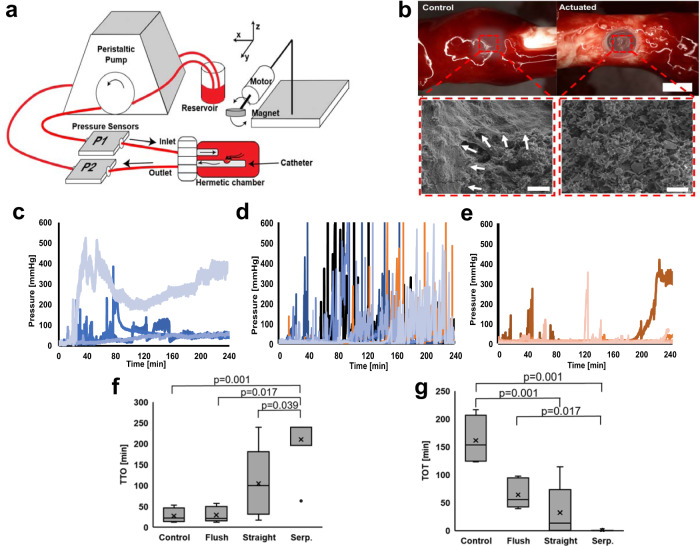
In vitro experimental setup and results. **a** Schematic of the bench-top blood circulation and magnetic actuation setup. **b** A representative visual comparison of control vs. actuated self-clearing catheter at the conclusion of the experiment. Scale bar = 1.5 mm. Inset: SEM images obtained from hematoma fragments of the control group. White arrows depict the robust fibrin network left unscathed and covering red blood cells. The hematoma fragments of the actuating group lack the fibrin network surrounding the red blood cells (*n* = 1, each). Scale bar = 10 μm. **c** Differential pressure (P2–P1) recording of all control catheters (*n* = 4). **d** Differential pressure (P2–P1) recording of all flushing catheters (*n* = 6). Note the frequent pressure spikes associated with each flushing. **e** Differential pressure (P2–P1) recording of all self-clearing catheters (*n* = 11). The figure includes data from five straight and six serpentine devices. **f** Comparison of time-to-occlusion (TTO) to reach 40 mmHg between the control catheters (*n* = 4), catheters that underwent flushing (*n* = 6), and self-clearing catheters. There were two versions of self-clearing catheters with either straight (*n* = 5) vs. serpentine flexure (*n* = 6) microactuators. One-way ANOVA with Tukey’s HSD analysis showed overall statistical significance between groups. The multiple comparisons between groups show significance as indicated. Each data point represents a different experiment using an independent sample. The box plot shows the interquartile range (IQR), the horizontal lines within the boxes are median. The *x* indicates the mean, and the whiskers represent the minima and the maxima of the data with outliers defined as data beyond 1.5× IQR. **g** Comparison of the total time over the threshold (TOT). One-way ANOVA with Tukey’s HSD analysis confirmed overall statistical significance between groups. The multiple comparisons between groups show significance as indicated. The sample size for each group is the same as in (**f**) and the box plots are defined in the same manner. Each data point represents a different experiment using an independent sample.

To demonstrate the blood-clot removal capabilities of our self-clearing catheters, we developed an in vitro circulation system^48^. Although several groups have reported a reduction of hematoma mass using a static condition^22,24^, none have demonstrated an effective clot prevention capability in a continuous flow environment, which is more physiologically relevant for our target application. Figure 3a shows the experimental setup, which mimics a fixed-volume ventricle that circulates sanguineous phosphate-buffered solution (PBS) as a CSF substitute. Using a peristaltic pump, we pumped diluted porcine blood (50:50 with PBS) out of the sealed chamber through different catheter designs. The blood–PBS mixture ratio was determined experimentally to reliably produce hematoma within 4 h. As the fluid flowed through the circulation system, we applied a time-varying magnetic field on all three groups: control, flushing, and self-clearing catheters (Supplementary Fig. 4). The flushed catheters were identical to control catheters but had a three-way stopcock valve attached to the line of the drainage device with one connector having a syringe with 2 mL of PBS. Once 5 min of pressure above the established threshold (40 mmHg) was reached, the valve was redirected to allow flow from the syringe, and the saline injection was introduced. After the full saline injection, flow in the circulation system was re-established. Flushing was repeated as many times as necessary in the 4 h experiment.

In general, the self-clearing catheters with integrated microactuators exhibited a smaller hematoma mass over their inlet pores compared to the flushing and control catheters (Fig. 3b). We also analyzed the effect of magnetic microactuation on the hematoma by analyzing the structure using an SEM (inset Fig. 3b). Utilizing a grading scale developed and analyzed by a board-certified clinical pathologist, we found that the fibrin mesh network significantly differed between the control and treatment devices (*p* < 0.001, Supplementary Fig. 5). The fragments from the treatment device displayed almost no fragments of fibrin, further confirming that our self-clearing catheters can break apart hematoma to allow drainage through the ventricular catheter.

We quantified the impact of magnetic microactuation by measuring the differential pressure between the inlet and the outlet during the experiment. Figure 3c–e shows differential pressure throughout the experiments. Without any blood in circulation, the differential pressure ranged between 5 and 15 mmHg, which is comparable to the normal intraventricular pressure of a patient. However, with blood, the control catheters exhibited an exceptionally high average pressure of 100 ± 111 mmHg, and the flushing catheters had an average pressure of 41 ± 10 mmHg, whereas the self-clearing catheters had an average pressure of 11 ± 31 mmHg. To compare all catheter groups, a threshold pressure of 40 mmHg was chosen because ICP exceeding this value is considered life-threatening^49–51^. The time-to occlusion (TTO) was defined by the time to reach this threshold. We postulate that TTO indicates how quickly the drainage system shows functional deterioration due to hematoma. For control catheters, the average TTO was 27 ± 18 min. For flushing catheters, the average TTO was 29 ± 18 min, which is similar to the control catheters. This was expected as both devices are single-pore catheters without the actuator. For the treatment group, the average TTO was delayed to 104 ± 86 min for straight devices and 211 ± 72 min for serpentine devices.

We also determined the total time over the threshold pressure of 40 mmHg (TOT). We postulated that the TOT indicates the resilience of the drainage system to combat blood-clot-induced failure. The average TOT for control devices was 162 ± 44 min during a 240 min experiment. The average TOT for the flushing catheters was 64 ± 25. In comparison, the average TOT was 33 ± 48 min for the catheters with straight devices and 0.3 ± 0.8 min for the catheters with serpentine devices. Figure 3f, g compares the TTO and TOT for each condition. Even at a lower threshold pressure (20 mmHg), our self-clearing catheters still exhibited a significantly better performance in terms of the TTO and TOT than the control and the flushed catheters (Supplementary Fig. 6).

In all four control catheters, we saw that the differential pressure remained above the threshold at the end of the circulation period, indicating robust and sustained obstruction by a hematoma (Fig. 3c). The catheters used for the flushing test had three out of six that remained above the pressure threshold, indicating large hematoma in the system was still present and was not absolved by the flushing. The average number of flushes per experiment run (*n* = 6) was 8 ± 3 resulting in high-pressure spikes outside of the pressure range of interest throughout the experiment time. Conversely, 8 out of 11 self-clearing catheters (5 straight and 6 serpentine devices) exhibited relatively lower pressure ( < 20 mmHg) at the end of the circulation (Fig. 3e). Compared to the control catheters, we also observed less frequent pressure spikes.

These results demonstrate that our self-clearing catheters can significantly delay occlusion due to hematoma and improve device reliability. Our experiments also show the cumbersome nature of having an experienced clinician be vigilant for obstruction and applying timely flushing with a risk of pressure spikes. In comparison, our self-clearing catheter can easily be actuated using external control with minimal risk for pressure spikes. Moreover, these results confirmed our hypothesis that the larger actuation deflection afforded by the serpentine flexure design could improve blood-clot removal capabilities. We suspect that the rapid translational motion of our microactuators causes localized shear at the catheter inlet, which exceeds the threshold above which hematoma becomes incapable to attach and accumulate on the catheter surface^52–54^. In future work, we may be able to use numerical evaluation to estimate the amount of shear stress our microactuators generate in a circulating flow environment.

### In vivo evaluation

**Fig. 4:**
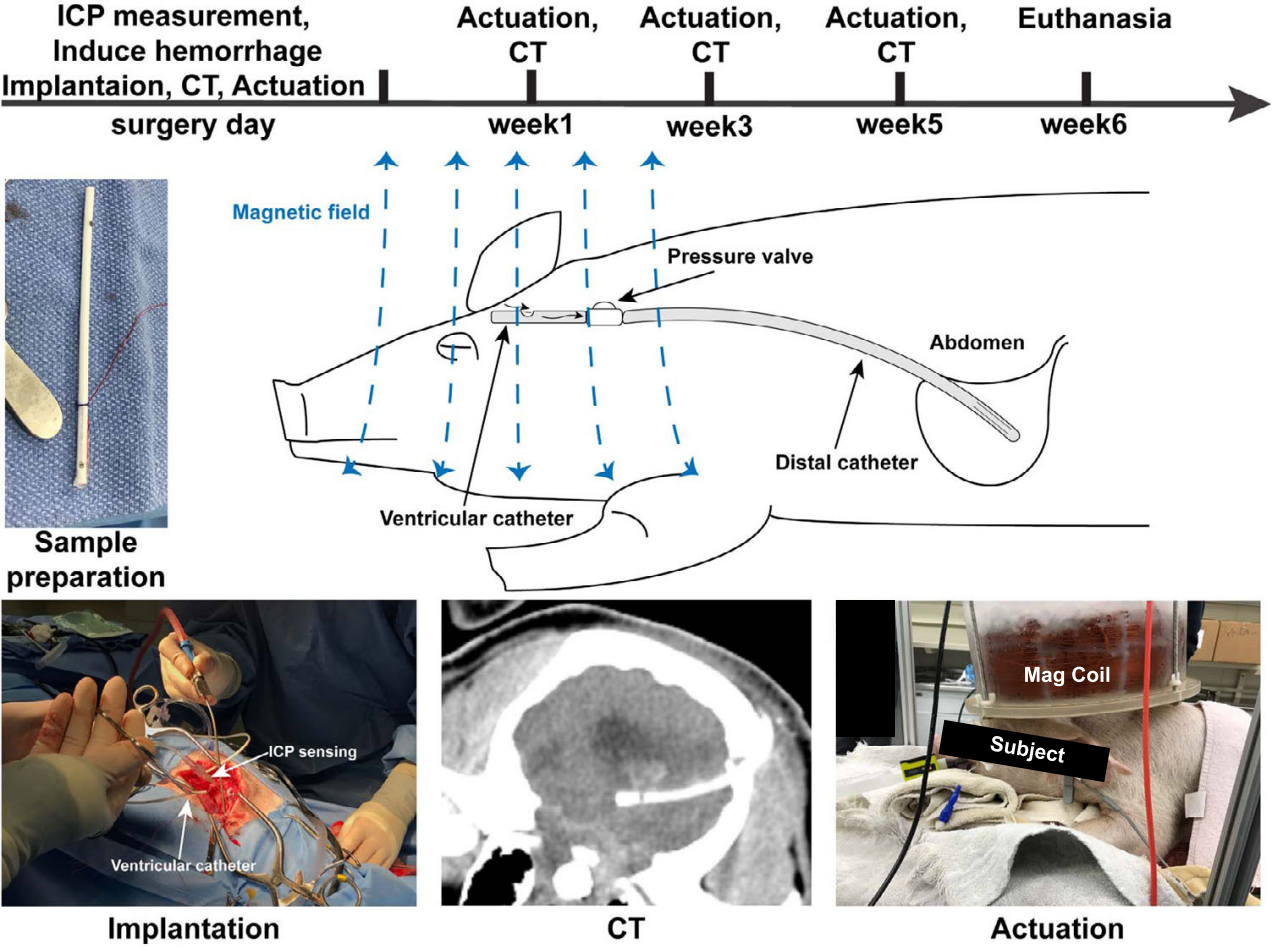
In vivo experimental timeline. The entire study is designed to last 6 weeks. On surgery day, we measured the intracranial pressure (ICP), induced the intraventricular hemorrhage (IVH), and implanted a ventriculoperitoneal shunt. The computed tomography (CT) scan was performed before and after the surgical procedure, to measure the ventricular size and confirm ventricular catheter placement. Magnetic actuation (30 min) was performed for the Treated group immediately afterward. At weeks 1, 3, and 5, each surviving animal was subjected to 30 min of magnetic field and CT scans. All surviving animals were terminated at week 6 for explantation and necropsy.

To verify the results from our in vitro experiment, we developed a porcine model of IVH and evaluated the impact of self-clearing catheters in vivo (Supplementary Fig. 9). Initially, we performed preliminary studies with six pigs to determine the amount of blood to be injected to cause large hematoma in the ventricle and sustained intracranial hypertension (Supplementary Fig. 10). As a result of these studies, we identified 10 ml of blood admixed with 140 units of thrombin immediately prior to injection as a reliable way to cause IVH and subsequent PHH. The blood and thrombin were injected into the right lateral ventricle in 3 equal aliquots. The timeline of the in vivo evaluation can be seen in Fig. 4.

A total of 13 pigs were used for the evaluation. The median weight was 28.0 ± 3.9 kg. The baseline ICP was 11 ± 5.2 mmHg. As the blood–thrombin injection was paused whenever ICP was ≥50 mmHg, the injection was performed over 2 to 15 min (median, 8 min). During injection of the third and final aliquot of blood, ICP would typically immediately increase above 50 mmHg with each injection of about 1 ml, then quickly fall (Supplementary Fig. 1). The ventricular catheter was placed 7.5 ± 7.0 min after the blood–thrombin injection was completed. A custom ventricular catheter with a single inlet pore was again used. ICPs reached a high but variable peak, fell quickly initially, then slowly declined to reach a plateau of around 13 ± 4 mmHg. The custom ventricular catheter was connected to a conventional one-way valve and peritoneal catheter to form a ventriculoperitoneal (VP) shunt. The fully implanted system was used in place of conventional EVD to mitigate the potential risk of surgical site infection and to allow free movement for the recovered animals over the 6-week experimental duration.

In all pigs, we used postoperative computed tomography (CT) to confirm the accurate intraventricular injection of blood and air. The post-shunting CT also confirmed the correct location of the ventricular catheter. In 1 Control and 1 Treatment pig, we determined that the only inlet pore had passed through the lateral ventricle and into the brain parenchyma. In both of these pigs, we performed a brief second surgery to retract the ventricular catheter and the subsequent CT confirmed the correct ventricular placement of the inlet pore.

**Fig. 5:**
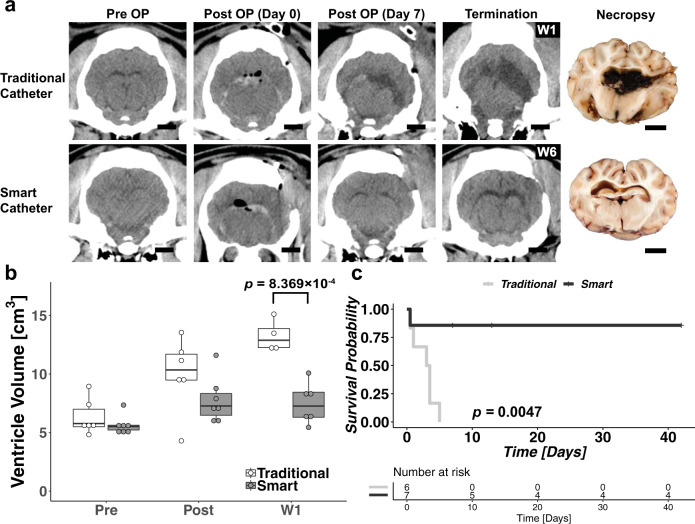
In vivo evaluation results. **a** Comparison of CT scans over the course of implantation for animals with traditional catheters (Control) and smart self-clearing catheters (Treated). Note the difference in ventricular volume before and after the surgery. There are signs of air pockets following the surgery (Post Op). After a week, the air pockets are resorbed but there is significant evidence of ventriculomegaly and hematoma in Control animals. The CT scan of the Control animal shows a significantly larger ventricle filled with hematoma compared to the Treatment animal. The photograph at necropsy also shows the enlarged ventricles. Scale bars = 1 cm. **b** Box plot of ventricle volume until week 1. The box plot shows the interquartile range, the horizontal lines within the boxes are median. The extended ventricle volume plot is available in Supplementary Fig. 7. Two-way ANOVA with Tukey’s HSD test indicated a significant difference in ventricle volume when treated with smart self-clearing catheters. Control animals with traditional catheters had a significant increase in ventricle volume by W1. **c** Kaplan–Meier survival plot with corresponding risk table. By week 1, the traditional shunt systems in all Control animals had failed whereas 80% of the shunt systems with self-clearing catheters remained obstruction-free with biweekly actuation. Supplementary Figure 8 shows the survival plot when infections are counted as failures.

All 6 Control pigs suffered sudden neurological decline and were found to have shunts obstructed with hematoma at necropsy. The sudden neurological decline in the Control pigs occurred 12 h to 5 d postoperatively (median, 3 d). Four of these animals became moribund within hours of the first sign of deterioration and were euthanized. Post-mortem CT revealed marked enlargement of the ventricles since the post-operative CT, transtentorial with or without cerebellar herniation, and continued correct ventricular location of the ventricular catheter without shunt displacement (Fig. 5a). The other two pigs showed a sudden, severe but non-fatal neurological decline. The ventricles were markedly larger on the next CT, and for the rest of the study. All explanted VP shunts in the control group showed evidence of hematoma inside their lumen (Supplementary Fig. 12). There were a total of eight hematomas in these six VP shunts (three ventricular catheters, three valvular, two distal catheters).

On the contrary, only 1 of the 7 Treatment pigs suffered neurological decline and was found to have a complete shunt obstruction with a hematoma at necropsy. This animal deteriorated 12 h post-operatively, rapidly became moribund, and was euthanized. This was the pig that had had a second surgery to slightly retract the ventricular catheter, resulting in delayed actuation. Upon post-mortem examination, we found further enlargement of the ventricles and persistence of intraventricular hematoma. Hematoma blocking the push connector (inlet for the valve) was found at necropsy (Supplementary Fig. 13a). The valve itself was filled with sanguineous CSF but was not obstructed. This was the only fatal hematoma in the seven VP shunts of the Treatment pigs. The ventricular catheters, valves, and distal catheters were otherwise patent without any evidence of obstructive hematoma (Supplementary Fig. 13b). These observations suggest that magnetic actuation may be able to preserve the patency of the downstream drainage path despite being at the proximal end of the ventricular catheter.

#### Infection

One Control pig suffered from non-fatal shunt obstruction with a hematoma on day 4, followed by dehiscence of the wound edges, post-operative infection, and euthanasia. Two Treatment pigs developed a fatal infection. One was euthanized on day 7 for peri-operative infection. The other rubbed his head on cage bars post-operatively. The skin edges were dehisced and were surgically re-closed on day 5 and again on day 7. Twelve days post-operatively his appetite decreased and 13 d post-operatively he was severely depressed, displayed nystagmus, and was euthanized.

In all three infected pigs, ventriculomegaly was seen at post-mortem CT, and during necropsy, we observed purulent discharge centered around the valve and surgical site and tracking down the ventricular catheter into the brain. In the two Treatment pigs, the purulent discharge obstructed the inlet pore. In the Control pig, there was also a hematoma obstructing the lumen of the ventricular catheter. After two infections in the first seven surgeries, we administered two peri-operative doses of florfenicol to each animal and added a subcutaneous muscle suture layer.

#### Ventricular volume

The postoperative CT in our animals showed significantly larger ventricles than prior to surgery in both groups (*p* = 0.002, Fig. 5a). By week 1, all air had been resorbed. In the Treatment group, all hematoma had been resorbed and ventricular size had non-significantly decreased. In the Control group, there had been a further enlargement of the ventricles and in some animals, hematoma persisted.

Figure 5b shows the change in ventricular volume until 1-week post-implantation. Two-way ANOVA showed statistical significance for both the device type and time. Specifically, the ventricular volume in the Control animals was significantly larger than in the Treatment group (*p* < 0.001). Moreover, the ventricular volume significantly increased before and after IVH and after a week of recovery (*p* < 0.001). Pairwise comparison showed that by W1, there was a significant difference in ventricular volume between the Control and the Treatment group (*p* < 0.001).

The data from W3 and W5 were omitted for statistical comparison because all Control shunts failed after W1. The change in ventricle volume over the entire implantation period is shown in Supplementary Fig. 7. Although the Treatment animals survived longer with our self-clearing catheters, the ventricle volume continued to increase, which may highlight the limitation of our study. This trend suggests that the self-clearing catheters are prolonging the functional lifetime but we may need to increase the actuation duty cycle to optimize treatment.

#### Kaplan–Meier survival analysis

We performed Kaplan–Meier analysis to evaluate the survivability of the shunt system due to hematoma, censoring the death of pigs due to infection (Fig. 5c). The median shunt survival in the Treatment group was 42 d and the median shunt survival in the Control group was 3 d. This difference was statistically significant (*p* = 0.0047), which further supports our hypothesis that the self-clearing ventricular catheters are more reliable and capable than existing drainage devices. We repeated the Kaplan–Meier analysis considering infection as a shunt failure, but the difference still remained statistically significant and the *p*-value unchanged (*p* = 0.0047, Supplementary Fig. 8), which further supports our hypothesis that the self-clearing catheters may improve the outcomes of IVH and PHH patients.

## Discussion

For the treatment of IVH, rapid removal of hematoma is critical in preventing further injury to the brain. Although EVDs may be used to facilitate hematoma clearance, we recognize that VP shunts are not used for immediate treatment of IVH. To demonstrate the long-term efficacy of our self-clearing catheters in vivo, however, it was necessary for us to use our device as a part of a fully implanted shunt system since it would have been nearly impossible to maintain the sterility of the transcutaneous EVD in our porcine model over 6 weeks.

It is also important to note that the prototype catheters we tested have a much higher risk of failure than a standard ventricular catheter. Conventional ventricular catheters typically contain 16 or more inlet pores (3 to 4 rows of 4 to 8 inlet pores)^55^. The self-clearing catheter we used had a single inlet pore to accelerate the occlusion, even though it is possible to integrate an array into the catheter (Supplementary Fig. 4c, Supplementary Movie 4). As such, the occlusion of this lone inlet pore would have led to an accelerated and complete device failure. Despite not having redundant pores for secondary flow, our results show that the single-pore device can maintain its patency due to periodic magnetic actuation.

The single inlet pore also increased the chance of inadequate surgical placement: the rows of inlet pores in conventional catheters maximize the likelihood that multiple inlet pores will be located in the lateral ventricle. In one Treatment pig, the post-operative CT indicated that this single inlet pore was too deep (passing through the ventricle and into brain tissue on the other side) and led to additional surgery and, most importantly, delayed actuation. This Treatment shunt was not actuated until after a second surgery to retract the ventricular catheter and was fatally obstructed in less than 24 h. Upon explantation, we found that the hematoma had passed the microactuator within the tip of the ventricular catheter and was at the junction of the ventricular catheter and valve, suggesting the first actuation may not have occurred until after overwhelming hemorrhage had already passed the actuator. All other Treatment shunts were first actuated soon after placement and subsequently remained free of hematoma obstruction throughout the 6-week study.

There was an additional risk of device failure as the catheter was placed directly into the ventricular hematoma, through the tract used for injection of blood, being inserted immediately after the injection was completed. Since the blood was mixed with the coagulant agent thrombin, we suspect that a mixture of CSF, blood, and thrombin immediately entered the catheter to begin coagulating. In current clinical practice, the presence of hematoma is a contraindication for shunting, which is typically recommended only when hydrocephalus has been diagnosed^6^. This extended waiting period can further exacerbate the brain injury due to the mass effect and result in a poor clinical outcome. Thus, we believe our work represents a significant improvement over the state-of-the-art in IVH treatment. Despite the added risk of device failure, our results highlight the robustness of our approach that enables earlier usage of drainage systems using our self-clearing catheters.

The effect of having a valve as a part of the VP shunt should also be carefully examined. Conventional EVDs typically do not have an implanted valve, which is often used to prevent over-drainage of CSF and siphoning effect for patients with VP shunts^56^. The presence of an implanted valve with small fittings that connects to the proximal catheter likely has exacerbated the shunt obstruction (Supplementary Fig. 12). However, our results showed that the actuated self-clearing catheter may also be able to prolong the lifetime of downstream components (i.e., pressure valve) as well, potentially by reducing the size of the blood clot that passes through the drainage pathway (Supplementary Figs. 5 and 13).

A commonly used valve in VP shunt surgery has a medium opening pressure (6–7 mmHg), which is within the range for normal ventricular pressure of 5–15 mmHg. We speculate that the low opening pressure may have increased the passage of hemorrhagic CSF through the VP shunt, as the coagulating fluid would have taken the path of least resistance rather than negotiated the ventricular system. Hemorrhage present within the ventricular system would be able to form a semi-permanent and permanent obstructive thrombus, rather than being dislodged by CSF flow. This would mandate the shunt system to remain functional to keep the patient alive, which further highlights the potential utility of our approach to maintain the patency of a drainage system using a self-clearing catheter. Although our experimental design did not explore the mechanism of clinical deterioration, it may be beneficial to characterize the change in CSF perfusion using a periodic CT perfusion or laser Doppler flow.

It is also important to note that the injection of blood in the in vivo experiment provides a substrate for bacterial growth. Typically, up to 20% of shunts fail due to infection-related issues^57^. This is exacerbated in neonates with PHH for whom 71% of shunt failures can be attributed to infection^58^. Although the scope of this work did not include the evaluation of infection prevention, there is evidence to suggest that the use of antibiotic-impregnated catheters can significantly reduce post-operative infections^58^. Thus, a logical next step may be to use a combination of microactuators with antibiotic or anti-inflammatory drug-eluting catheters to ensure that our self-clearing catheter can not only accelerate hematoma clearance but it can provide robust protection against infection-related device failures^31^.

We also compared the performance of our self-clearing catheter against the common clinical practice of using saline flush to remove drainage device obstruction. Although saline flushes are effective in removing the obstruction, each flushing caused a potentially dangerous level of a pressure spike, and the catheter was rapidly re-occluded, which necessitated more frequent flushing. Pressure spikes notwithstanding, the flushed catheters remained above the safe pressure range for longer periods than our self-clearing catheters. Our results highlight the clinical challenge of having a caretaker constantly monitoring patient ICP to ensure that the drainage device performs reliably until the hematoma clears. In comparison, our approach may be used with minimal user input using an overhead electromagnet that can be placed over the patient’s head. Furthermore, by optimizing the actuation duty cycle, we believe it may be possible to improve the performance and utility of drainage devices with our self-clearing smart catheters.

Overall, our experimental results demonstrate a novel non-pharmaceutical approach of using magnetic microactuator-enabled smart catheters to treat IVH in situ using externally applied magnetic fields. We demonstrated that these magnetic microactuators can expedite the removal of blood from the ventricle, maintain a lower ventricle volume, and increase the survival rate of IVH-suffering animals. Although additional work is needed, we believe these types of magnetic microactuator-embedded smart catheters can potentially improve the lifetime and the reliability of chronically implanted catheters that are critical in a number of neurological, urological, cardiovascular, and other drug delivery applications.

## Methods

### Device fabrication

The microactuators were fabricated in a standard cleanroom environment. Starting from a 100 mm p-type single-side polished silicon wafer (Silicon Quest, San Jose, CA, USA), 50 nm of silicon dioxide was deposited by plasma-enhanced chemical vapor deposition (Axic, Milpitas, CA, USA) to function as a release layer. Next, a layer of polyimide PI-2525, HD Microsystem, Parlin, NJ, USA) was spun coated at 1750 rpm and cured in a nitrogen oven up to 350 °C to create a final thickness of 11 μm. A 50-nm-thick Cr was evaporated (Airco E-Beam Evaporator, Livermore, CA, USA) as the etch mask for the polymer layer. Cr mask was photo-patterned using AZ9260 (Microchemicals, Germany) and etched using a Cr etchant (Cr-16, KMG, Fort Worth, TX, USA). The polyimide structural layer was etched using O_2_ plasma at 20 sccm, 50 mTorr and 150 W RF power (Advanced Oxide Etcher, Surface Technology System, Newport, UK). Cr mask was then removed using Cr-16.

Next, 100 nm of Au was sputtered (PVD, Wilmington, MA, USA) globally as a conduction layer and 200-μm-thick electroplating mold was photo-patterned using a negative photoresist (BPN-65A, Rohm Haas, Marlborough, MA, USA). The nickel ferromagnetic elements were in a 2L plating solution maintained at 60 °C with a 40 mA direct current for 4 h. The plating solution contained 1 M nickel sulfamate, 0.4 M boric acid, and 10 g sodium dodecyl sulfate. The electroplated elements varied from 80 to 130 μm in thickness depending on the actuator geometry. Afterward, the exposed Au conduction layer was stripped using a wet etchant (GE8148, Transene, Danvers, MA, USA). The microactuators were released from the wafer using a buffered oxide etchant (Fisher Scientific, Waltham, MA, USA). To create the self-clearing catheter, the released sample was rolled and inserted into a catheter lumen (Model G0664, Cook Medical, Bloomington, IN, USA)^27^.

### Finite element analysis

COMSOL Multiphysics (V5.0, COMSOL, Inc., Burlington, MA, USA) was used for the finite element analysis of device deflection and stress distribution. Each device design was configured to have the material property of polyimide with a density of 1300 kg/m^3^, Young’s modulus of 2.45 GPa, and a poison ratio of 0.3. Using the solid mechanic’s module, each flexure was fixed on one end and a vertical point load ranging from 1 to 100 μN was applied to the free end to evaluate the static deflection and the stress distribution. Lagrange strain was used to account for the large flexure deformation.

### Static and dynamic mechanical evaluations

To characterize the static response, each sample was positioned along the long axis of a bespoke solenoid electromagnet (cylindrical permalloy core, 1-in-diameter, and 6-in-tall with 300 turns) and down to the edge of a glass slide. The distance between the sample and the electromagnet surface was kept at 7 mm to minimize the magnetic flux density gradient (<0.1 T/m) while maintaining an adequate amount of parallel magnetic field strength. A DC power source (PWS2326, Tektronix, Beaverton, OR, USA) was used to supply the current to the electromagnet. The amount of magnetic flux density at the position of actuators was measured using a gaussmeter (8010, F.W. Bell, Milwaukie, OR, USA). During actuation, the electromagnet along with actuator positioning glass slides was placed horizontally and the amount of deflection angle was optically measured using a digital SLR camera (Canon 50D, Huntington, NY, USA). The magnetic flux density was varied from 0 to 22 mT for the straight beamed device and from 0 to 17 mT for the serpentine device.

To quantify the dynamic response, each sample was mounted on a glass slide and placed in a beaker filled with DI water. The device was then actuated using a bespoke iron-core electromagnet that was driven using a Rigol DG1022 (Beaverton, OR, USA) signal generator coupled with an AE Techron 7224 DC enabled AC amplifier (Elkhart, IN, USA) between 1 and 100 Hz at 15 mT. The dynamic motion of the microactuator at each frequency interval was captured using a Chronos 1.4 high-speed camera (Kron Technologies, Vancouver, B.C., Canada). The amplitude of the actuator was then quantified using image tracking (ImageJ, v4).

### In vitro evaluation

A 50 ml glass bottle (Duran, Fisher Scientific, Waltham, MA, USA) was used to mimic the ventricular chamber. The bottle was sealed using a screw cap with two through holes allowing placement of the inlet and outlet tubing (L/S 14, Masterflex, Cole-Parmer, Vernon Hills, IL, USA). A piece of a central venous catheter (Model G0664, Cook Medical, Bloomington, IN, USA) was attached to the outlet inside the glass bottle chamber. The through-holes on the screw cap were sealed using a silicone adhesive to fix the tubings in place. For the catheters used for flushing, a three-way stopcock (Masterflex, Fisher Scientific, Waltham, MA, USA) was introduced to the outlet of the ventricle chamber. A 10 mL syringe with 2 mL of 1× PBS (Fisher Scientific, Waltham, MA, USA) was attached to one of the inlets of the valve. A variable speed peristaltic pump (7523-50, Masterflex, Cole Parmer, Vernon Hills, IL, USA) was used to drive fluid into the chamber at 1.4 ml/min, about twice the rate of average cerebral spinal fluid production in humans^56,59^. Two pressure sensors (PRESS-S-00, PendoTech, Princeton, NJ, USA) were connected to the inlet and the outlet tubes.

A mixture of porcine blood and 1× PBS (Fisher Scientific, Waltham, MA, USA) at 50:50 vol was used to mimic hemorrhagic CSF. The fresh blood of euthanized pigs was mixed with 10 USP units of heparin/ml and stored in a refrigerator at 4 °C for 1 week prior to the experiment. At the time of testing, protamine sulfate (Fisher Scientific, Waltham, MA, USA) was added (10 mg per 100 USP heparin) to reverse the effects of anti-coagulant and to facilitate blood clot formation^60^. We studied the rheological behavior of our blood mixture using a cone and plate rheometer (Supplementary Fig. 5a). Steady shear tests were performed at 37 °C following the protocol from Alves et al.^61^. Briefly, we used a controlled stress rheometer ARG2 (TA Instruments, New Castle, DE, USA) with a 20-mm-diameter cone with a 1° angle attachment and 0.040 mL of the blood mixture for testing steady shear rates ranging from 0.1 to 500 s^−1^. Our results demonstrate the established shear thinning behavior of blood. The typical viscosity of blood ranges from 3.5 to 5.5 cP depending on the hemodynamic conditions^62^. At the highest shear rate (500 s^−1^) the apparent viscosity of the blood mixture was 12.33 cP, which is a higher than normal range found in humans. Since conventional catheter occlusion is unpredictable with a wide range of time to failure^63,64^, a layer of fibrin matrix gel was applied on the catheter surface surrounding the inlet pore to further promote blood clots attachment. The fibrin gel was made from a mixture of fibrinogen (38 mg/ml) and thrombin (37 mg/ml) (Thermo Fisher Scientific, Waltham, MA, USA)^65–67^.

Each device was subjected to 4 h of circulation in the circulating setup during which the differential pressure was recorded continuously (12 samples/min)^25^. For Control (*n* = 4) and Flush (*n* = 6) groups, single-pore catheters without any microactuators were tested. For Treatment groups, self-clearing catheters with either straight (*n* = 5) or serpentine flexure designs were tested (*n* = 6). During each experiment, the time-varying magnetic field was applied using a permanent magnet (McMaster-Carr, Elmhurst, IL, USA) affixed to a DC motor from 20 mm away spinning at 8 Hz. The magnetic performance of this motorized setup is shown in Supplementary Fig. 14. Using the differential pressure recording, the time to reach a threshold pressure (i.e., time to occlusion, TTO) and the time over a threshold pressure (TOT, 40 mmHg) was calculated. The TTO and TOT for different conditions were compared using one-way ANOVA with Tukey’s HSD post-hoc analyses with *p* < 0.05 as statistical significance (Microsoft Excel, v. 16).

The ultrastructure of hematoma fragments was analyzed for the integrity of the fibrin fiber network. All blood–PBS solution exiting the VC was collected directly into a 200 mL 4% glutaraldehyde and 4% paraformaldehyde fixative for preparation for SEM hematoma fragment analysis. The blood–PBS fixed solution was prepared for SEM analysis^68^. Samples were washed with three cycles of PBS (5 min each). Secondary fixation was performed with 1% osmium tetroxide for 1 h. The fragments were rinsed in de-ionized water three times at 5 min per cycle. All samples were dehydrated in a series of ethanol solutions of increasing concentration for 10 min each (10%, 30%, 50%, 70%, 85%, two times at 95%, and three times at 100%) followed by critical point drying. Finally, samples were placed on aluminum mounts and carbon-coated prior to SEM. A grading scale was developed to semi-quantitatively measure the samples (Supplementary Fig. 5). Samples were analyzed and scored by a board-certified (American College of Veterinary Pathologists) clinical pathologist. All control (*n* = 36) and treatment (*n* = 18) samples were analyzed and scored by a board-certified (American College of Veterinary Pathologists) clinical pathologist.

### In vivo evaluation

All surgical procedures on mice were performed following all ethical regulations as approved by the Purdue Animal Care and Use Committee under protocol number 1602001368. For the in vivo evaluation, 13 cross-bred domestic swines weighing 25.0–31.6 kg were used (Fig. 4). We used the time-to-occlusion results to estimate the sample size of *n* = 3 with the power of 0.8 and *α* of 0.05. We doubled this number for a target of *n* = 6 to ensure that we have enough power to account for potential variations in animal testing. We also performed a post hoc analysis using the hazard ratio of 0.08 (Supplementary Table 3). Using the hazard ratio, we calculated the acceptable sample size of *n* = 4 per group with the following parameters: *β* = 0.8, *α* = 0.05, median survival time = 7 d, and the planned average length follow-up = 42 d.

Each pig was pre-medicated with 0.2 mg/kg midazolam (Hikma Pharmaceuticals USA, Eatontown, NJ, USA) and 0.03 mg/kg dexmedetomidine (Zoetis US, Parsippany, NJ, USA) and induced with isoflurane delivered by face mask until tracheal intubation was possible. Anesthesia was continued with isoflurane delivered in 100% oxygen. Mechanical ventilation, pulse oximetry, capnography, indirect blood pressure measurement, temperature measurement, left jugular central line placement, and intravenous fluid therapy (10 mL/kg/h) were performed. Analgesia was provided by the pre-operative transdermal application of 2.7 mg/kg fentanyl solution (4-d expected activity) (Elanco Greenfield, IN). Intramuscular injection of 5 mg/kg ceftiofur (7-d expected activity) was used for antibiotic treatment.

To induce IVH, a right side subtemporal craniectomy was performed using a standard aseptic technique. To measure intraventricular ICP, a 1.25-in. 18-gauge intravenous cannula was directed through the intact dura mater into the right lateral ventricle. The correct ventricular location was confirmed by drainage of CSF. Non-compliant tubing (pre-filled with saline) was connected tightly and attached to a transducer and monitor. To inject the blood–thrombin mixture, a second 1.25-inch 18-gauge intravenous cannula was then placed into the lateral ventricle, more rostrally. The drainage of CSF again confirmed accurate placement. A total of 10 ml of autologous blood and 140 units of thrombin was injected through this cannula as determined using preliminary studies. Blood was drawn from the jugular catheter using the sterile technique in three equal aliquots. Each aliquot was briefly agitated with thrombin in a syringe and then injected. Each time ICP rose above 50 mmHg, the injection was paused until ICP was <50 mmHg. This second cannula was then withdrawn.

The puncture hole in the dura mater was slightly enlarged with a #11 scalpel blade, and the ventricular catheter was inserted through the tract that had been used for blood injection. In six pigs, a Control VP shunt was placed. The ventricular catheter was custom-made for this study using a modified central venous access device (Cook Model G0664, Bloomington, IN, USA), with only one single inlet pore. In seven pigs, a Treatment shunt was placed. The custom-made ventricular catheter had a microactuator placed at the single inlet pore^27^. The correct ventricular location was confirmed by rapid drainage of sanguineous CSF. ICP recording was continued until 30 min after the placement of the ventricular catheter.

The ventricular catheter was anchored to the skull by a pre-placed polydioxanone suture (Ethicon, Somerville, NJ, USA) through the periosteum and loose fascia. It was connected to a pre-placed low-pressure valve (Integra Lifesciences, Plainsboro, NJ, USA) and distal catheter which had been filled with saline, creating a VP shunt. The valve was anchored to the periosteal tissue of the posterior skull by a suture that encircled the connection of the ventricular catheter to the valve. The valve was pumped, and the functionality of the VP shunt was confirmed by sanguineous CSF entering the valve and saline escaping from the distal catheter. The distal catheter was placed in the peritoneal cavity and the paracostal incision closed. The fascia of the temporalis muscle was closed with a simple continuous polydioxanone suture. The skin was closed with simple continuous subcuticular and intradermal poliglecaprone sutures (Ethicon, Raritan, NJ, USA), followed by the application of skin glue.

CT was performed prior to and immediately following surgery (day 0). In the Treatment group, actuation was performed immediately after post-operative CT for 30 min using a bespoke electromagnet that was powered using a Rigol DG1022 (Beaverton, OR, USA) signal generator coupled with an AE Techron 7224 (Elkhart, IN, USA) DC enabled AC amplifier. (Supplementary Fig. 15). The electromagnet has a maximum magnetic field strength of 50 kA/m which is orders of magnitude below the necessary magnetic field strength for transcranial magnetic stimulation^69^. Pigs were then recovered from anesthesia. Repeat CT was performed at weeks 1, 3, and 5. Pigs were again premedicated with 0.2 mg/kg midazolam and 0.03 mg/kg dexmedetomidine, then had oxygen and isoflurane delivered by face mask throughout the CT. In the Treatment group, repeat actuation was performed immediately after each CT. The first CT was 5–7 d post-operatively so that the first actuation was performed on day 5 or 6 in the Treatment group.

The size of the ventricles was measured using a DICOM viewer (Osirix Lite 10.0.1, Pixmeo SARL Bernex, Switzerland). The ventricle (i.e., region of interest) from each CT scan was outlined manually by three measurers. The volume of the outlined region of interest was calculated by the DICOM viewer and compared (Supplementary Fig. 16). One-way ANOVA with Tukey’s HSD post hoc analysis showed no statistical significance between the three measurers (*p* = 0.095) using RStudio (v. 1.1.419, Boston, MA, USA). To compare the difference in ventricle volumes in the Treatment vs. the Control group, two-way ANOVA was performed to see the effects of time and the type of catheters using RStudio. Post hoc pairwise comparisons were made using Tukey’s HSD test.

Kaplan–Meier analysis of shunt survival was used to compare Treatment and Control groups using RStudio. Obstruction of the shunt by hematoma was considered an event. Both for fatal and non-fatal shunt obstructions causing a neurological decline, we noted the increased size of the lateral ventricles on CT and we verified the shunt obstruction by hematoma at necropsy when these animals were sacrificed at day 42. Death of the pig due to infection was censored.

### Reporting summary

Further information on research design is available in the Nature Research Reporting Summary linked to this article.

## Supplementary information

Supplementary Information

Description of Additional Supplementary Files

Supplementary Movie 1

Supplementary Movie 2

Supplementary Movie 3

Supplementary Movie 4

Reporting Summary

## Data Availability

The authors declare that all other relevant data supporting the findings of the study are available in this article and in its Source Data file. Source data are provided with this paper.

## Source data

Source Data
