# Supplementary material for: Application of magnetically actuated self-clearing catheter for rapid in situ blood clot clearance in hemorrhagic stroke treatment
Source: Nat Commun. 2022 Jan 26;13:520. doi: 10.1038/s41467-022-28101-5 (PMC8791973; doi:10.1038/s41467-022-28101-5)
Supplement: Supplementary file 1 — Supplementary Information [file 41467_2022_28101_MOESM1_ESM.pdf]

Application of magnetically actuated self-clearing catheter for rapid in situ blood clot clearance in hemorrhagic stroke treatment

Qi Yang,[1,2,3,4,7] Angel Enriquez,[1,2,3,7] Dillon Devathasan,[5] Craig A. Thompson,[5] Dillan Nayee,[1,2] Ryan Harris,[1,2] Douglas Satoski,[1,2] Barnabas Obeng-Gyasi,[1,2] Albert Lee,[6] R. Timothy Bentley,[5] Hyowon Lee,[1,2,3*]

[1]Weldon School of Biomedical Engineering
[2]Center for Implantable Devices
[3]Birck Nanotechnology Center
[4]School of Electrical and Computer Engineering
Purdue University, West Lafayette, IN 47907, USA

[5]College of Veterinary Medicine
Purdue University, West Lafayette, IN 47907 USA

[6]Goodman Campbell Brain and Spine
Indianapolis, IN 46202 USA

[7]These authors contributed equally.
[*]To whom correspondence should be addressed; E-mail: hwlee@purdue.edu.

Supplementary Materials

Supplementary Tables

Supplementary Table 1: Magnetic microactuator design dimensions

Type	Straight	Serpentine
Beam length [μm]	600	3000
Beam width [μm]	75	55
Beam thickness [μm]	11	11
Tip volume [$\times 10^{-3}$mm^3]	46	40
Ni thickness [μm]	130	80
Ni long axis [μm]	775	905
Aspect ratio	5.9	11.3

Supplementary Table 2: Measured dimensions of fabricated magnetic microactuators ($\pm s.d.$, $n = 3$, independent samples)

Type	Straight	Serpentine
Beam length [μm]	595±1.5	2676±18
Beam width [μm]	74.7±0.4	56.8±1.1
Beam thickness [μm]	11.25±0.08	11.16±0.06
Tip volume [$\times 10^{-3}$mm^3]	46±2.4	43±1.6
Aspect ratio	5.9±0.29	10.6±0.38

Supplementary Table 3: Post-hoc hazard ratio for in vivo evaluation ($n = 13$, independent samples).

Characteristic	HR[1]	95% CI[1]	p-value
Group	0.08	0.01, 0.68	0.0221

[1]HR = Hazard Ratio, CI = Confidence Interval

Supplementary Discussion

Analytical model of magnetostatic response

Magnetic properties characterization After magnetic actuators were fabricated, the magnetic properties of electroplated nickel were evaluated using a SQUID magneto meter (MPMS-3, Quantum Design, Inc., San Diego, CA, USA). Saturation magnetization, coercivity, remanence, susceptibility and overall hysteresis of the electroplated nickel elements were used to predict magnetic torque produced and to provide guidance for optimizing the applied magnetic field. A sample of serpentine flexure device was cut so that only nickel structure and cantilever remained. The long axis of the sample was aligned to the moving direction of the test stage and the external magnetic field direction. For measuring saturation, the sample was tested from -1 to 1 T. High field saturation loop indicated that the electroplated nickel saturates at 200 kA/m with saturation magnetization of 0.75 T (Supplementary Fig. 1a). A low field hysteresis loop was also captured from -30 to 30 kA/m to get a better resolution on critical points across axes. Minimum hysteresis was observed and Supplementary Fig. 1b shows the coercivity of 2 kA/m and remanence of 0.037 T. The magnetization varied almost linearly with respect to the field strength at low field range. The magnetic susceptibility (χ) was 11.3.

The AC susceptibility was tested to characterize how well the magnet responds with the external sinusoidal excitation. At 0 T DC bias, the sample was exposed to 1 mT sinusoidal magnetic filed. The driving frequency varied from 7 to 550 Hz and the corresponding AC susceptibility (χ', χ'' [emu/Oe]) was measured. The magnitude (M/H, unitless) and the phase angle (degrees) were then converted. The results indicated less than 2% variation in susceptibility and less than 0.7° phase lag across the tested frequency range. This suggests that the direction of magnetization turns almost instantaneously with respect to the external sinusoidal signal while the magnitude of magnetization remains constant within our actuation frequency

(<100Hz).

Magnetostatic torque In the presence of uniform magnetic field, magnetic material experiences magnetic torque given by $\vec{\tau_m} = \mu_0 v \vec{\mathrm{M}} \times \vec{\mathrm{H}}$ where $\vec{\mathrm{M}}$ is magnetization, μ_0 is magnetic permeability of free space, v is volume and $\vec{\mathrm{H}}$ is external magnetic field. For hard magnetic material (i.e., permanent magnet), the magnetic torque can easily be estimated by applying the saturation magnetization value if the variation of external field is within coercivity. Ferromagnetic material such as electroplated nickel, however, is magnetically soft. Both the direction and the magnitude of magnetization vary as a function of the applied magnetic field amplitude and the magnetic anisotropy. An improved model based on Judy [1] and Abbott [2] is therefore developed to better describe the magnetic torque and the motion of magnetic actuator with polyimide flexure. If a uniform magnetic field H_a is applied along one axis, the internal magnetic field H experiences by nickel is given by $H = H_a - N_d M$. Demagnetization factor N_d describes how strong the internal field is reduced along the given axis due to the geometric anisotropy[3]. The demagnetization factors have been solved analytically for ellipsoid geometry and the values for other geometries have been extensively studied [4, 5, 6, 7]. Here, the demagnetization factor of an oblate spheroid was used. The value of N_d depends on the aspect ratio between lengths of long axis and thickness (Table 2) With a higher aspect ratio, we can expect the N_d become larger for the specified axis. Introducing the magnetic susceptibility $\chi = \frac{M}{H}$ and acknowledging the fact that M reaches to magnetic remanence M_r when H_a is removed, the magnetization has the form of:

$$M = \frac{\chi}{1+\chi N_d} H_a + M_r. \tag{1}$$

In 3 dimensional cartesian coordinates, magnetic torque is therefore described by:

$$\vec{\tau_m} = \mu_0 v \begin{bmatrix} \frac{\chi}{1+\chi N_x} H_x + M_r \\ \frac{\chi}{1+\chi N_y} H_y + M_r \\ \frac{\chi}{1+\chi N_z} H_z + M_r \end{bmatrix} \times \begin{bmatrix} H_x \\ H_y \\ H_z \end{bmatrix} \tag{2}$$

During magnetic actuation the applied field was positioned in a way that there was no horizontal component perpendicular to the length of cantilever (Supplementary Fig. 2a, Supplementary Fig. 3a, Supplementary Fig. 3a). Only x and z components of magnetization remain and the angle between the net magnetic field H and the shortest axis of the magnet is θ. Assuming the sample is previously magnetized along the long axis x and M_r remains only along x direction, then the magnetic torque can be calculated by:

$$\vec{\tau_m} = \mu_0 v \begin{vmatrix} \hat{a_x} & \hat{a_y} & \hat{a_z} \\ \frac{\chi H sin\theta}{1+N_x\chi} + M_r & 0 & \frac{\chi H cos\theta}{1+N_z\chi} \\ H sin\theta & 0 & H cos\theta \end{vmatrix} \tag{3}$$

$$\vec{\tau_m} = \mu_0 v(-\frac{\chi H^2 sin\theta cos\theta}{1+N_x\chi} - M_r H cos\theta + \frac{\chi H^2 sin\theta cos\theta}{1+N_z\chi})\hat{a_y} \tag{4}$$

and the rotation produced by mechanical torque is along y axis as shown in equation 4. Notable differences between this model and model from [1, 2] are the inclusion of observed magnetic remanence and the finite susceptibility. This mathematical model suits our application for the case when the device operates at linear region in the hysteresis loop.

Static deflection angle of polyimide-based magnetic actuator can be calculated by balancing the magnetic torque from the ferromagnetic element and mechanical torque from the flexure [8]. Considering a magnetic field fixed in z direction and structural plate is only rotating along x-z plane, the relation $\tau_k = k\theta$ was previously used for predicting amount of mechanical torque required to produced transverse bending angle θ of a straight beam with rectangular

cross section[9, 10]. A modified relation is used to account for an initial angle θ_i produced from residual stress of a polyimide film from thermal curing[11].

$$\tau_k = k(\theta + \theta_i); k = \frac{Ewt^3}{12L} \tag{5}$$

E is modulous of elasticity of polyimide, w, L, t are cantilever width, length and thickness respectively. By equating equation 4 and equation 5, static deflection angle θ as a function of magnetic field strength H in z direction is calculated. Supplementary Fig. 2 shows improvement of static deflection prediction.

Dynamic response of magnetic actuator

AC mechanical response of magnetic actuator with serpentine cantilever was characterized to determine the optimum actuation frequency. The device was fixed in a transparent evaporation dish filled with DI water. A bespoke electromagnet was placed above the device for supplying time-varying sinusoidal magnetic field. A high speed camera (Chronos 1.4, Kron Technologies, Inc. Barnaby, BC, Canada) was positioned horizontally to image and record vertical deflection of the ferromagnetic element (Supplementary Fig. 3a). Actuation frequencies from 3.5 to 60 Hz were applied and for each frequency the magnitude of vertical H field was adjusted at 15 mT, the amount determined from the DC analysis. High speed footages were analyzed for each actuation frequency frame by frame. Position of the outer edge of the ferromagnetic element on the actuator was measured. The vertical deflection range was calculated by subtracting a highest and a lowest point ever reached by the actuator. The results indicate a response similar to behavior of a low pass filter. Deflection range maintained at around 1.7 mm until 15Hz and then quickly reduced to 60 μm at 60 Hz due to liquid damping (Supplementary Fig. 3b-c). The average speed during one actuation cycle was also calculated for each frequency. A peak speed of 4.5 cm/s was produced between 10 to 15Hz. In order to produce large deflection and maintain

high speed at the same time, an actuation frequency of 15Hz or smaller should be considered.

Magnetic flux density during the *in vitro* experiment

A spacial and time varying magnetic field generation setup was made to deliver maximum torque for each actuation cycle. Typically, a multi-coil system is used to provide such H pattern in AC. However, since our frequency of interest is under 100Hz, a spinning neodymium magnet was utilized for its simplicity. A one-inch wide, half-inch-thick circular neodymium disk magnet was housed inside a 3D printed fixture connected to a driving shaft driven with a DC motor (Supplementary Fig. 3a). The neodymium magnet was oriented in a way that H field was minimum in the y-axis. The direction of H changed in a circular fashion in x-z plane each cycle. Each actuator was placed approximately 3.5 cm below and 2 cm away from the Nd magnet such that the maximum magnetic flux density was 14 mT (Supplementary Fig. 14). The effective frequency of 8 Hz (240 rpm from motor) was selected for maximum deflection range and motor stability.

Supplementary Figures

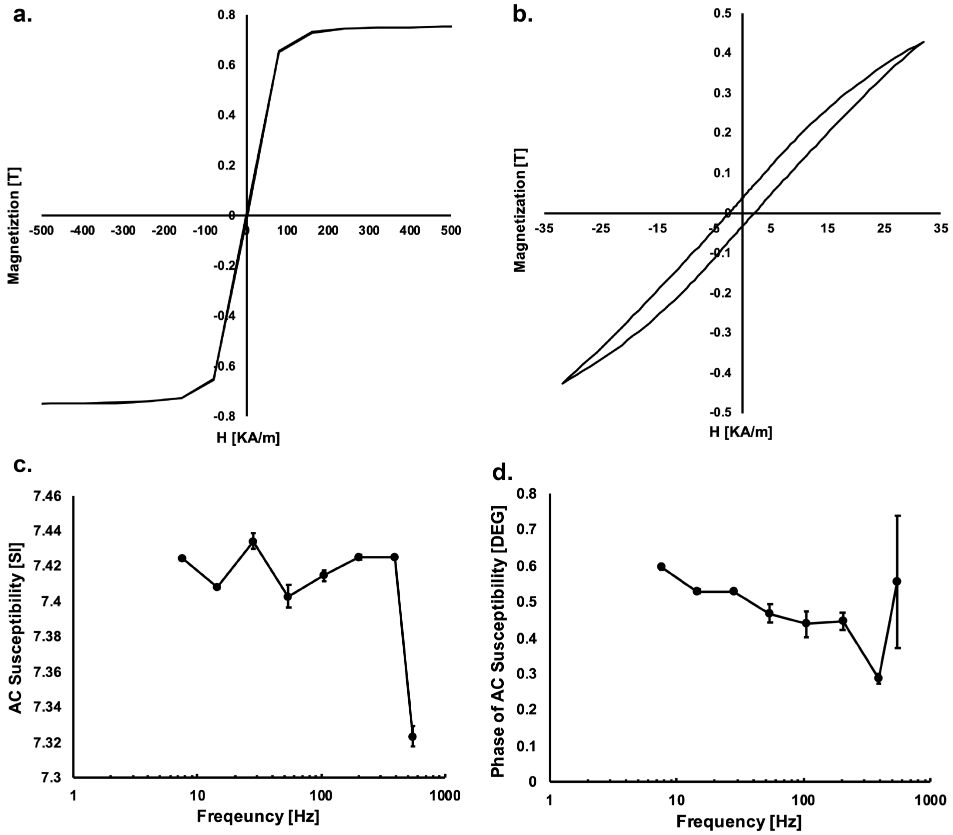

Supplementary Figure 1: Magnetic characterization of electroplated nickel. (a) M-H loop within high field range. (b) M-H loop within low field range. (c) Magnitude of AC magnetic susceptibility from 7 to 550 Hz. Data presented as mean ± standard error ($n = 3$). Each data point represents an independent sample. (d) Phase of AC magnetic susceptibility from 7 to 550 Hz. Data presented as mean ± standard error ($n = 3$). Each data point represents an independent sample.

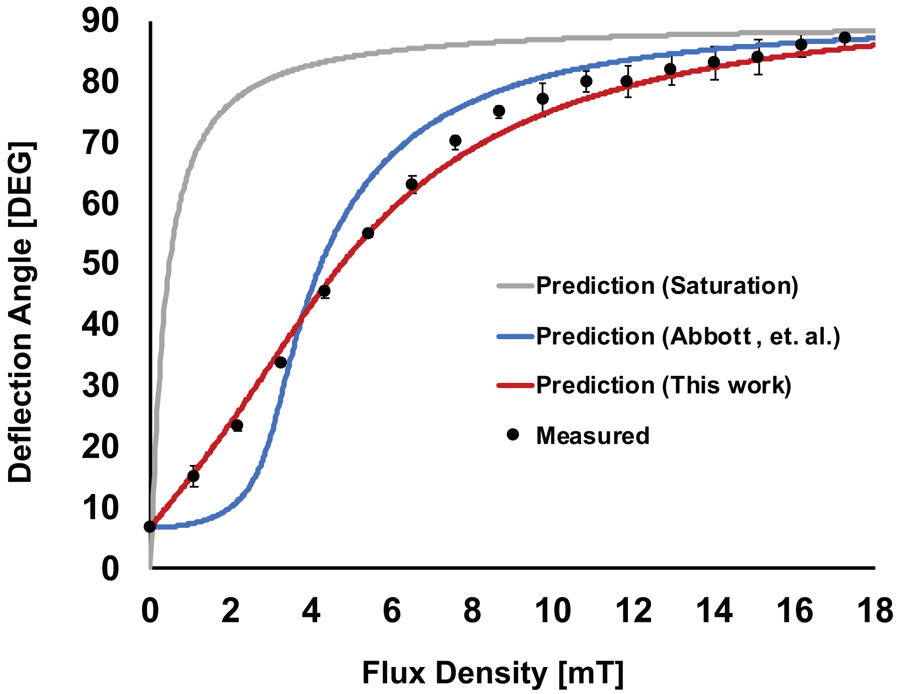

Supplementary Figure 2: Measured static deflection angle in comparison to various saturation models. Data presented as mean ± standard deviation ($n = 3$). Each data point represents an independent sample.

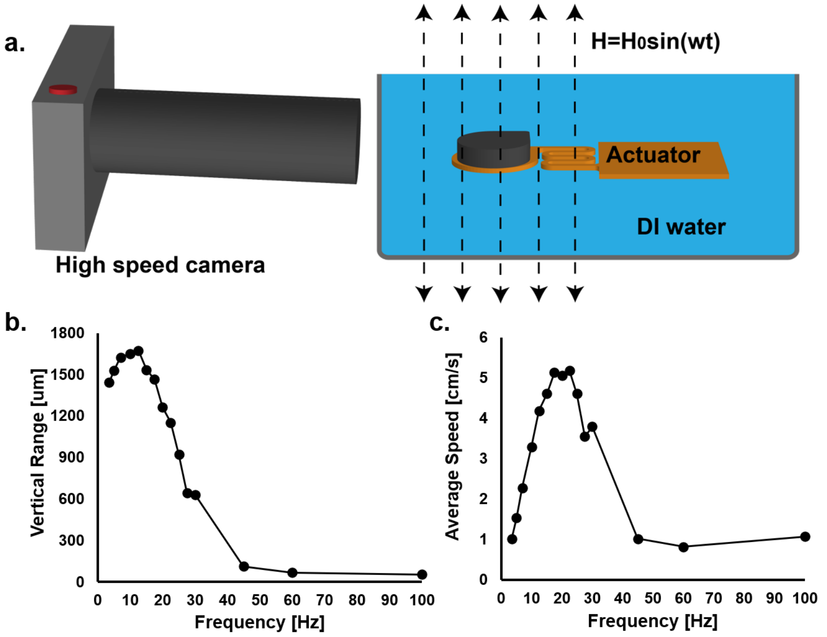

Supplementary Figure 3: Dynamic response measurements. (a) Schematic illustration of dynamic response of the serpentine actuator in water. (b) Vertical deflection range as a function of frequency. (c) Average speed at outer edge of the actuator as a function of frequency.

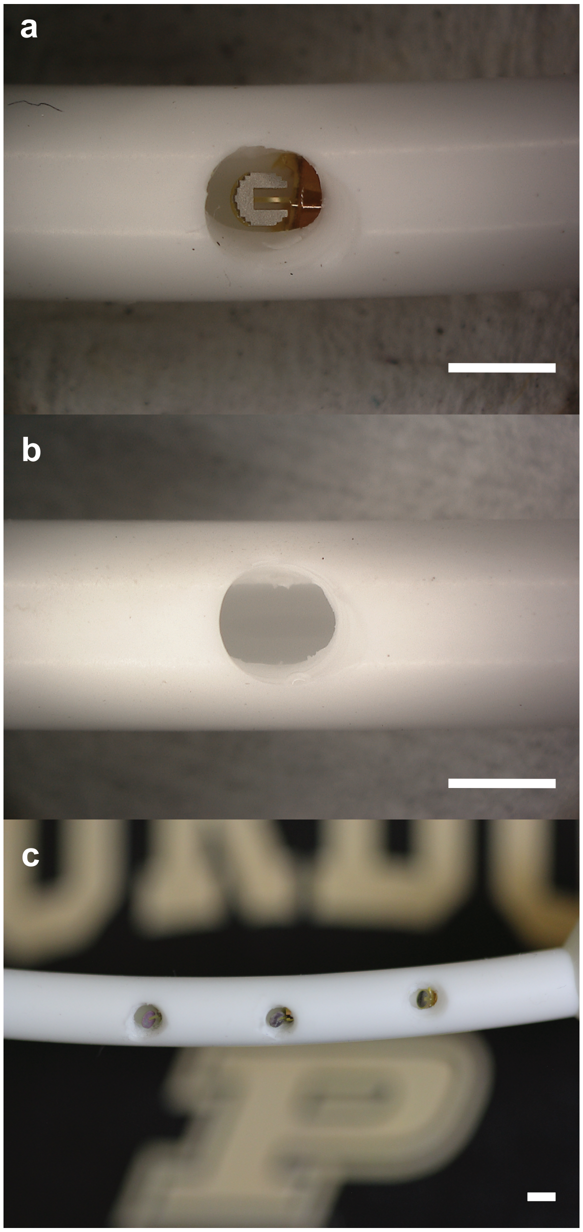

Supplementary Figure 4: Images of a self-clearing catheter vs. a control catheter. (a) A sample of treatment catheter integrated with magnetic microactuator. (b) A sample of control catheter. (c) Multi-pore catheter with integrated microactuators. Scale bar = 1.5mm

a.

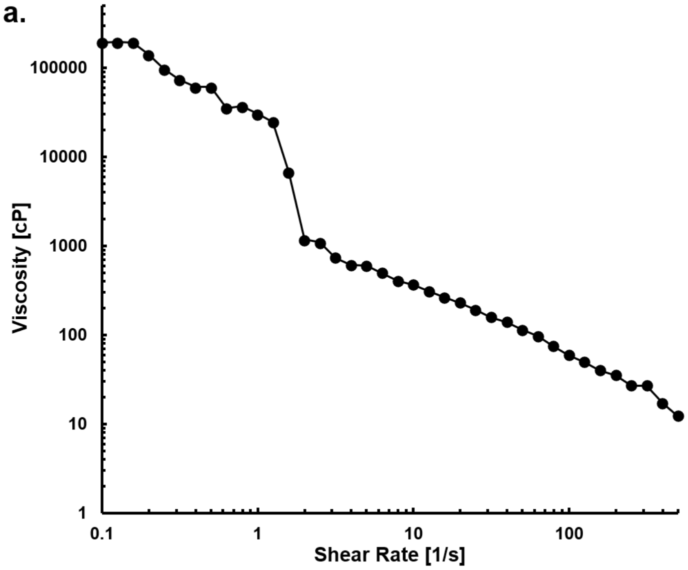

b.

Grade	Comment
0	Fibrin mesh network easily seen with numerous long fibrils present. Erythrocytes trapped within the fibrin mesh.
1	Fibrin network observed, with loose arrangement of occasional long fibrin fibrils noted.
2	Few individualized to rarely cohered/adhered fibrin fibrils noted.
3	Rare and/or short fragments of fibrin seen cohered to one another or to the surface of erythrocytes
4	Extremely rare or no fibrin fragments observed

c.

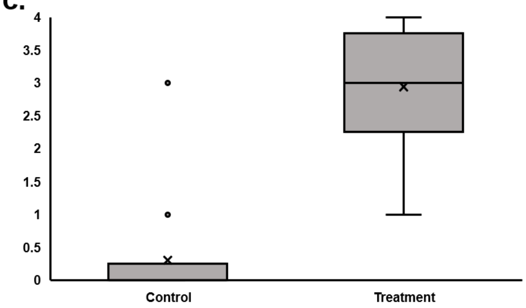

Supplementary Figure 5: (a) Flow curve of the blood mixture used in our in vitro evaluation. (b) Fibrin network grading scale. (c) Fibrin grading from the control and the treatment (self-clearing) catheters obtained from clinical pathologist analysis (Control: $n = 36$, Treatment: $n = 18$).

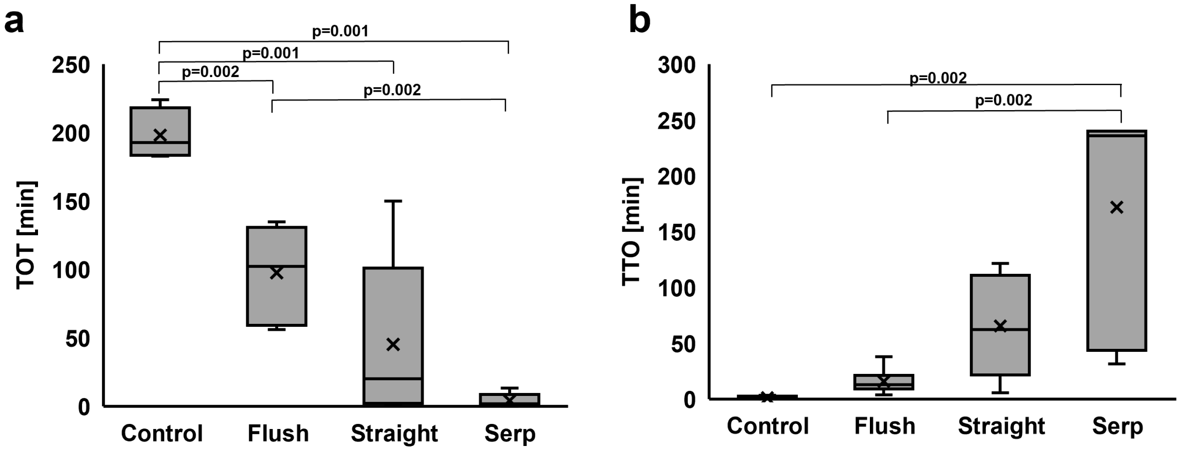

Supplementary Figure 6: (a) Comparison of time to occlusion (TTO) to reach 20 mmHg between the control catheters, catheters that underwent flushing, and self-clearing catheters. (b) Comparison of the total time over threshold (TOT) at 20 mmHg. The boxplots show the interquartile range (IQR), the horizontal lines within the boxes are median. The x indicates the mean, and the whiskers represents the minima and the maxima of the data with outliers defined as data beyond $1.5 \times$ IQR. Each data point represents different experiment using an independent sample. The sample sizes are the same as shown in main Figure 3.

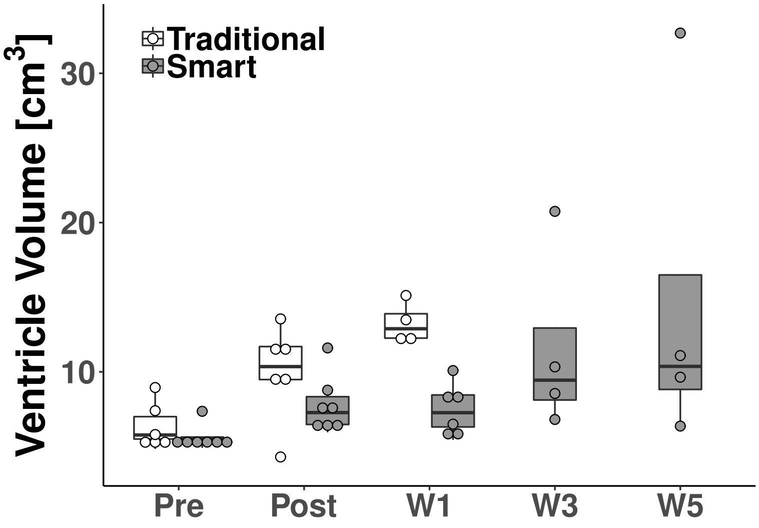

Supplementary Figure 7: Box plot of ventricle volume for the entire duration of chronic experiment. Note that the ventricle volume continued to increase in Treatment group, which may suggest a more frequent actuation may be needed over an extended period. Each data point represents different experiment using an independent sample.

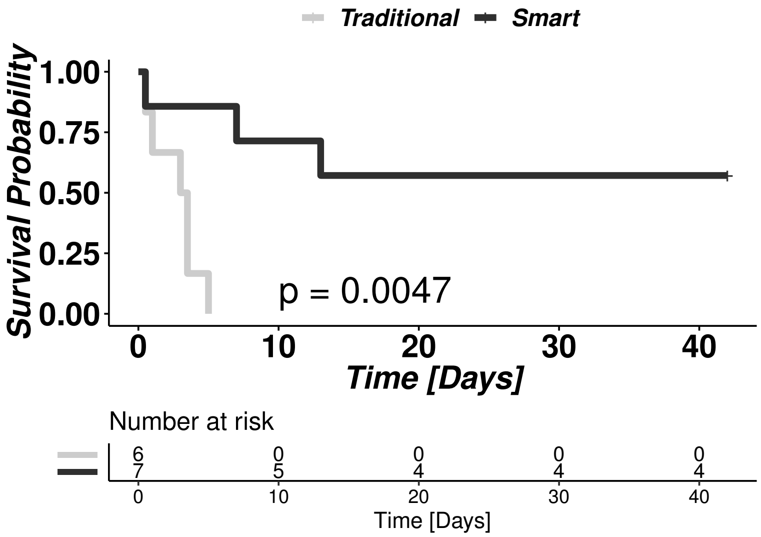

Supplementary Figure 8: Kaplan-Meier survival plot with corresponding risk table when infections are counted as failures. By week 1, the traditional shunt systems in Control animals had failed where as more than 50% of the shunt systems with self-clearing catheters remained hematoma-free with biweekly actuation. The *p*-value did not change and the difference remained statistically significant.

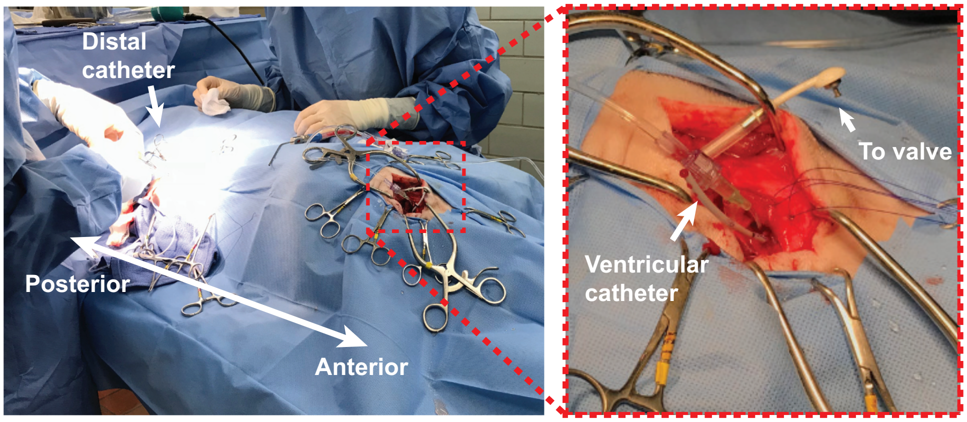

Supplementary Figure 9: Sterile surgical setup for the intraventricular hemorrhage and the implantation of a shunt system in a porcine model. Inset: A zoomed image of the surgical site showing the ventricular catheter prior to being connected to a pressure valve.

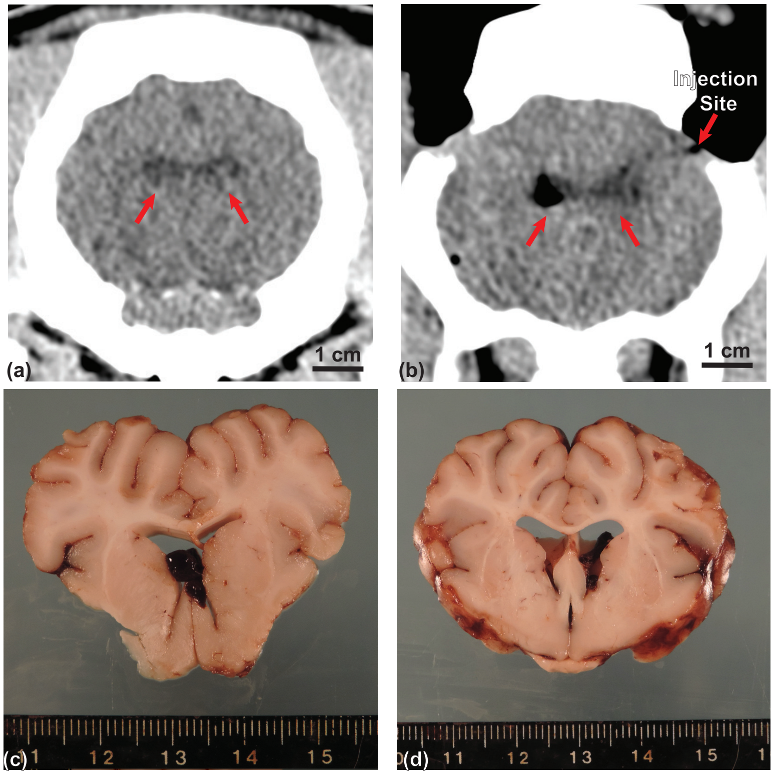

Supplementary Figure 10: Induction of intraventricular hemorrhage. The (a) pre- and (b) post-operative CT scans of the animal that shows grossly enlarged ventricle. Note the visible tract into the right lateral ventricle that was used to inject autologous blood. (c) Post-mortem sections of cerebral cortex showing enlarged right ventricle. Note the deviated septum pellucidum and the presence of large hematoma in the right ventricle. (d) Bilateral enlargement in the body and the temporal horn of lateral ventricles. Note the hemorrhage present in the left ventricle and in the cerebral aqueduct.

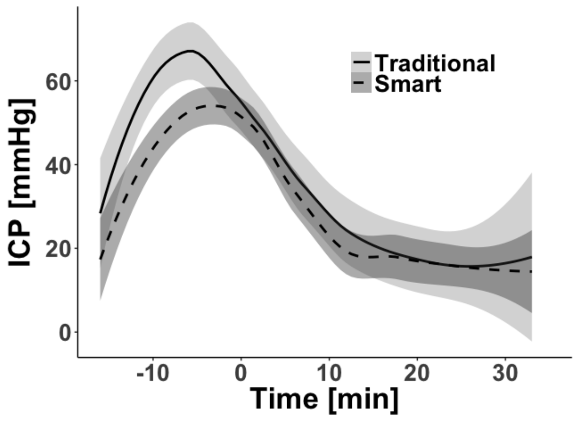

Supplementary Figure 11: Measurement of intracranial pressure (ICP) during injection of autologous blood. To induce intraventricular hemorrhage, 10 ml of autologous blood was injected. Time = 0 indicates the point when the entire volume was injected. Note that the ICP remained abnormally high regardless of which catheter type was used. Control ($n = 4$), Smart Catheter ($n = 6$) Data presented as the mean (line) and 95% confidence band (shade).

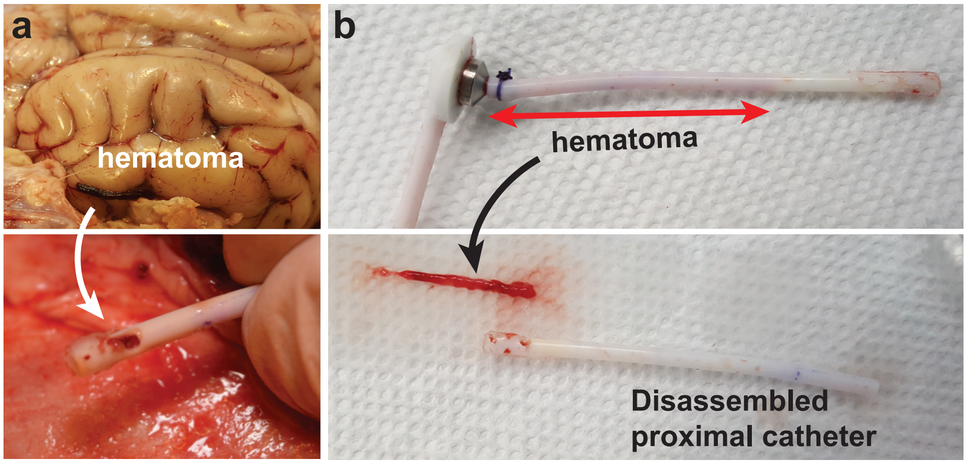

Supplementary Figure 12: Photographs of explanted failed control catheters. (a) The explanted control ventricular catheter showed complete blockage in its inlet poor with hematoma. When pulled out of the brain, a long track of blood clot was pulled out from the catheter. (b) When the shunt system was dissembled, a fresh hematoma was found, which suggests that this device was completely occluded.

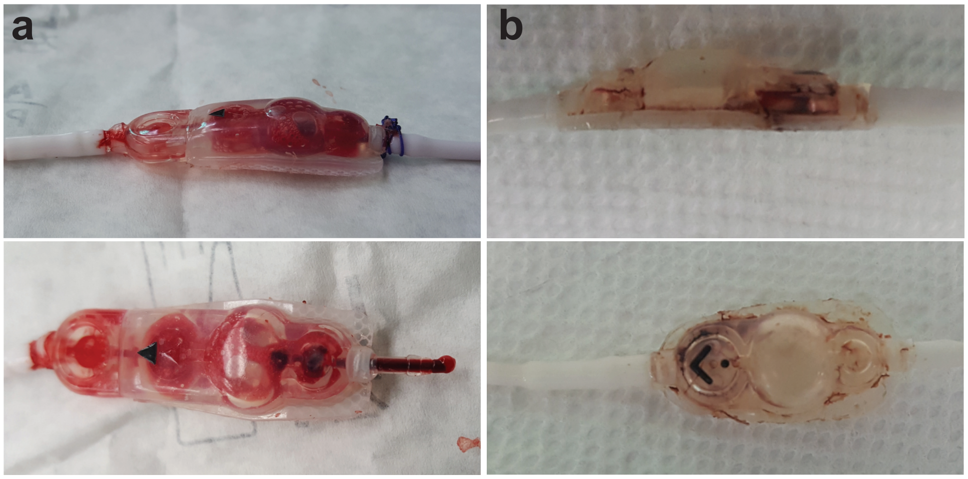

Supplementary Figure 13: Explanted pressure valves for treated groups. (a) Photograph of the explanted pressure valve from the Treatment animal that perished on the day of surgery. We found that the push connector portion was blocked with hematoma. This animal had a revision surgery due to initial mis-position of the catheter into the cortex. (b) Photograph of an explanted pressure valve from a Treatment animal that survived the entire 6 week duration. We saw that the valve was patent without any evidence of large hematoma.

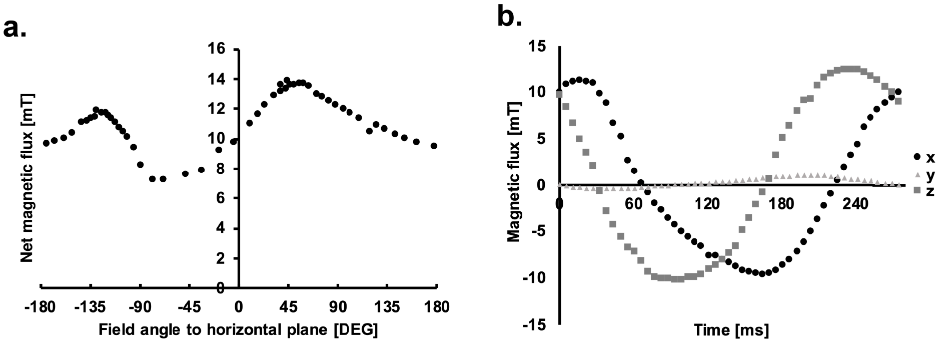

Supplementary Figure 14: Measured magnetic flux density from the custom magnetic field generator. (a) Net magnet flux density actuator experienced vs. the horizontal plane. (b) Magnetic flux density components from 3 axis that a device experiences during one period of revolution.

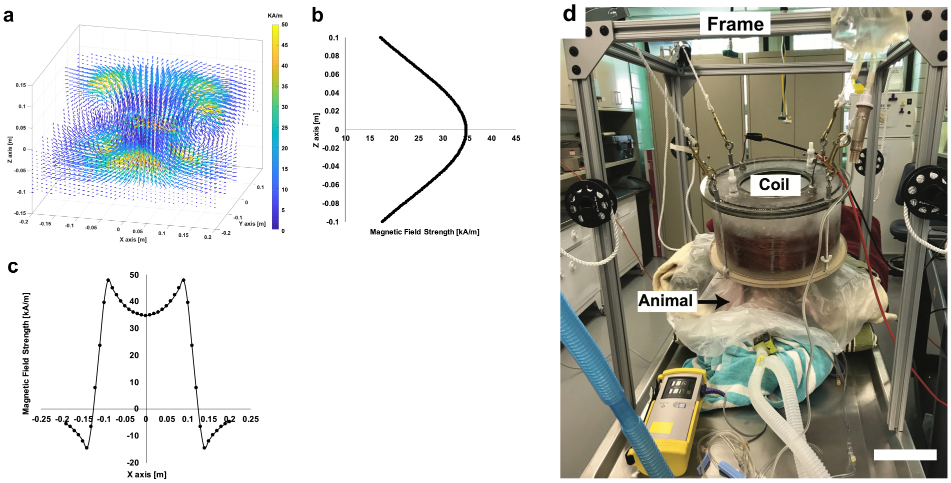

Supplementary Figure 15: A custom electromagnet apparatus for *in vitro* experiment. (a) 3-D magnetic field line distribution of the electromagnetic coil. (b) Magnetic field strength distribution along the z-axis. (c) Magnetic field strength distribution along x axis at midway from the top and bottom and midway to a side. (d) A photograph of the suspended electromagnet placed over the anesthetized animal. The coil is a multilayer air-core solenoid. Inner diameter = 10cm. Outer diameter = 14 cm. Height = 11 cm. Insulated AWG 12 copper wire was used. The coil has 518 turns and 14 layers. Current = 17 A. Scale bar = 15 cm.

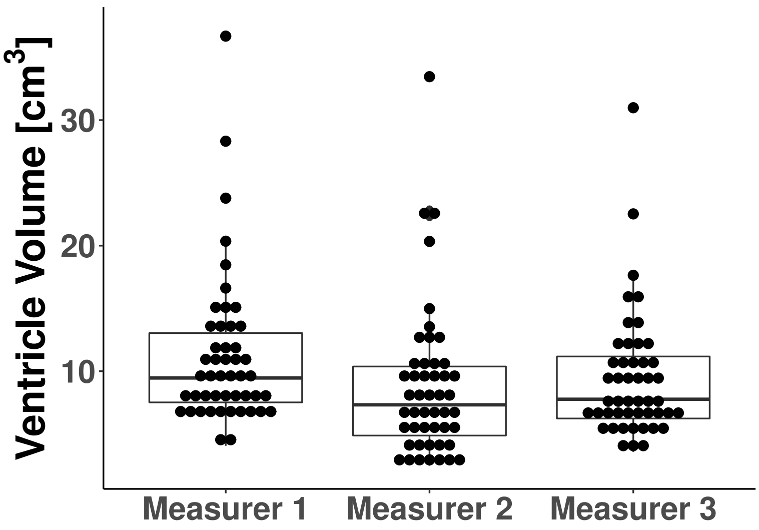

Supplementary Figure 16: Comparison of ventricle volume measured by three separate individuals. The box plot shows the interquartile range (IQR), the horizontal lines within the boxes are median. One-way ANOVA indicated no significant differences between the three measurers ($p = 0.095$). Each data point represents measurement by each measurer from different experiment using an independent sample.

Supplementary References

[1] J. W. Judy and R. S. Muller, “Magnetically actuated, addressable microstructures,” *Journal of Microelectromechanical systems*, vol. 6, no. 3, pp. 249–256, 1997.

[2] J. J. Abbott, O. Ergeneman, M. P. Kummer, A. M. Hirt, and B. J. Nelson, “Modeling magnetic torque and force for controlled manipulation of soft-magnetic bodies,” *IEEE Transactions on Robotics*, vol. 23, no. 6, pp. 1247–1252, 2007.

[3] B. D. Cullity and C. D. Graham, *Introduction to magnetic materials.* John Wiley & Sons, 2011.

[4] J. Osborn, “Demagnetizing factors of the general ellipsoid,” *Physical review*, vol. 67, no. 11-12, p. 351, 1945.

[5] A. Aharoni, “Demagnetizing factors for rectangular ferromagnetic prisms,” *Journal of applied physics*, vol. 83, no. 6, pp. 3432–3434, 1998.

[6] D.-X. Chen, J. A. Brug, and R. B. Goldfarb, “Demagnetizing factors for cylinders,” *IEEE Transactions on magnetics*, vol. 27, no. 4, pp. 3601–3619, 1991.

[7] D.-X. Chen, E. Pardo, and A. Sanchez, “Fluxmetric and magnetometric demagnetizing factors for cylinders,” *Journal of Magnetism and Magnetic Materials*, vol. 306, no. 1, pp. 135–146, 2006.

[8] H. Lee, K. Kolahi, M. Bergsneider, and J. W. Judy, “Mechanical evaluation of unobstructing magnetic microactuators for implantable ventricular catheters,” *Journal of Microelectromechanical Systems*, vol. 23, no. 4, pp. 795–802, 2014.

[9] R. J. Roark, *Roark’s Formulas for Stress and Strain.* New York: McGraw-Hill, 1989.

[10] Q. Yang, H. Park, T. N. Nguyen, J. F. Rhoads, A. Lee, R. T. Bentley, J. W. Judy, and H. Lee, “Anti-biofouling implantable catheter using thin-film magnetic microactuators,” *Sensors and Actuators B: Chemical*, vol. 273, pp. 1694–1704, 2018.

[11] G. Elsner, “Residual stress and thermal expansion of spun-on polyimide films,” *Journal of applied polymer science*, vol. 34, no. 2, pp. 815–828, 1987.
