# Supplementary material for: Application of magnetically actuated self-clearing catheter for rapid in situ blood clot clearance in hemorrhagic stroke treatment
Source: Nat Commun. 2022 Jan 26;13:520. doi: 10.1038/s41467-022-28101-5 (PMC8791973; doi:10.1038/s41467-022-28101-5)
Supplement: Supplementary file 2 — Description of Additional Supplementary Files [file 41467_2022_28101_MOESM2_ESM.pdf]

Description of Additional Supplementary Files

File Name: Supplementary Movie 1
Description: High speed video of serpentine microactuator movement.

File Name: Supplementary Movie 2
Description: High speed video of a blood clot being dislodged from the catheter pore.

File Name: Supplementary Movie 3
Description: Actuator dynamic response with image tracking.

File Name: Supplementary Movie 4
Description: A multi-pore catheter in action.
